# Non-surgical Rhinoplasty (NSR): A Systematic Review of Its Techniques, Outcomes, and Patient Satisfaction

**DOI:** 10.7759/cureus.50728

**Published:** 2023-12-18

**Authors:** Dhuha S Al-Taie, Esraa M AlEdani, Jahnavi Gurramkonda, Shaan Chaudhri, Amina Amin, Binay K Panjiyar, Tuheen Sankar Nath

**Affiliations:** 1 Otolaryngology-Head and Neck Surgery, California Institute of Behavioral Neurosciences & Psychology, Fairfield, USA; 2 Dermatology and Internal Medicine, California Institute of Behavioral Neurosciences & Psychology, Fairfield, USA; 3 Neurological Surgery, California Institute of Behavioral Neurosciences & Psychology, Fairfield, USA; 4 Psychiatry, California Institute of Behavioral Neurosciences & Psychology, Fairfield, USA; 5 General Surgery, California Institute of Behavioral Neurosciences & Psychology, Fairfield, USA; 6 Internal Medicine, California Institute of Behavioral Neurosciences & Psychology, Fairfield, USA; 7 Surgical Oncology, Tata Medical Centre, Kolkata, IND

**Keywords:** nose job, cosmetic procedure, filler around the nose, hyaluronic fillers, liquid rhinoplasty, facial cosmetic surgery, filler injection, rhinoplasty surgery, ent surgery, ent procedures

## Abstract

Surgical rhinoplasty (SR), commonly known as nose job, is a widely practiced cosmetic surgery globally, aimed at addressing diverse aesthetic and functional concerns related to the nose. In recent years, non-surgical rhinoplasty (NSR) has gained popularity due to advanced techniques involving hyaluronic acid (HA) dermal fillers, offering advantages such as affordability, reduced side effects, and faster results. However, concerns persist about the suitability of dermal fillers for nasal anatomy and potential complications, prompting this comprehensive review. This study systematically evaluated the techniques, fillers, safety, and patient satisfaction associated with NSR, with the intent of providing valuable insights for clinicians and patients considering NSR or SR for improved aesthetic outcomes. The literature search, following Preferred Reporting Items for Systematic Reviews and Meta-Analyses (PRISMA) criteria, yielded 16 relevant studies from an initial pool of 1002 articles. These studies covered various aspects of NSR, including techniques, complications, limitations, and positive results. In conclusion, NSR appears to be a quick and safe option for addressing minor nose shape issues, particularly through the use of HA fillers, but further discussion and standardization are necessary to address risks and limitations. A randomized controlled trial (RCT) using photographic evidence could significantly propel the progress of this evolving treatment. RCTs offer an optimal method to assess NSR's adverse effects and overall outcomes by allowing controlled comparisons between treatment and control groups. This approach minimizes biases and generates reliable statistical data, which is critical for evaluating safety, efficacy, and potential risks, thereby guiding informed clinical decisions.

## Introduction and background

Surgical rhinoplasty (SR), also known as nose job, is one of the most common cosmetic surgical procedures performed globally, with more than 350,000 operations carried out annually in the United States [1]; it is intended to reshape and improve the appearance of the nose. This surgical procedure can address a variety of issues, such as the repair of nasal asymmetries, the diminution of a pronounced hump, the refining of the nasal tip, and modifications to the size and form of the nostrils. In addition to being performed for cosmetic purposes, rhinoplasty can also be used to improve breathing and fix problems with the nasal passages' functionality. With advancements in surgical techniques and technology, SR has become a highly personalised procedure, tailored to the unique goals and anatomy of each patient [2]. 

Although the International Society of Aesthetic Plastic Surgery suggested that the nose is an area that is more amenable to surgical procedures, the introduction of advanced dermal filler techniques may favour non-surgical rhinoplasty (NSR) [3]. Due to their lower cost, lower risk of side effects, and shorter duration, NSRs have become widely favoured by people who desire to improve their nose shape. NSR can be used for shaping, fixing, and contouring the nose. The most common procedure performed with dermal fillers is fixing and creating a smooth nasal bridge, also known as dorsal augmentation [3].

Considering the nose anatomy and its thin skin compared to other facial regions, there is still a lack of information regarding the best treatments, techniques, and indications for safe and effective nasal filler reshaping [4]. Studies have shown that if dermal fillers are accidentally injected into the facial vasculature, it might result in complications including eyesight loss, brain damage from a cerebrovascular accident, or necrosis of the adjacent skin and facial tissues. Thus, extreme caution must be used when applying fillers around the nose [5]. These major adverse effects have led to questions over whether NSR is a suitable alternative to SR.

Because surgeons and patients need accurate information to make decisions about the safest and most suitable therapy for their unique needs, it is important to investigate which procedure produces superior aesthetic results and higher patient satisfaction. To create a useful comparison between the two types of aesthetic treatment, this study of the literature will attempt to take stock of what is currently known about NSR and the different types of fillers and treatments. The study will also focus on patients’ feedback and satisfaction regarding the use of NSR as a replacement for SR.

## Review

Method

In this systematic review, a PICO (population, intervention, comparison, and outcome) statement was formulated to provide a structured framework for guiding the methodology section. The patient population (P) under study comprises individuals with minor nose deformities who are reluctant to undergo SR. The primary focus of the investigation (I) is to evaluate the advantages and positive results associated with NSR. This will be compared (C) to the conventional SR approach. The primary outcome (O) to be assessed is the determination of whether NSR represents a viable alternative to SR. 

The Preferred Reporting Items for Systematic Reviews and Meta-Analyses (PRISMA) criteria were followed for conducting this literature review [6]. The PubMed research engine was used to create advanced Medical Subject Headings (MESH) with the following string: ((Nose job OR rhinoplasty OR nose reconstruction surgery OR (“Rhinoplasty/adverse effects"[Majr] OR “Rhinoplasty/classification"[Majr] OR “Rhinoplasty/history"[Majr] OR “Rhinoplasty/standards"[Majr])) AND (Cosmetic nose job OR nose fillers OR Cosmetic nasal fillers OR Cosmetic nose enhancement OR Liquid rhinoplasty)). 

Before the article screening and data extraction process, inclusion and exclusion criteria were set; these are illustrated in Table 1.

**Table 1:** Inclusion and exclusion criteria

Inclusion criteria	Exclusion criteria
Studies that include results, patient satisfaction, and potential hazards of rhinoplasty and cosmetic nose surgery.	Studies that are not written in full text.
In terms of trialled studies, studies that have a properly defined research population of individuals who have undergone non-surgical rhinoplasty treatment.	Studies that are in a different language aside from English.
Studies written in English language only.	Studies covering other aesthetic procedures.
Studies that are written in full text.	Studies that do not meet the quality check scales.
Studies that were published in 2018 and later.	

The Google Scholar search engine was used to conduct a second search for articles discussing SR and NSR. In this review, all publications on SR and NSR were incorporated. In this study, only studies that were available in the English language and released after 2018 were included. The chosen time frame aims to integrate publications featuring the most recent advancements, methodologies, and updated information, ensuring the comprehension of current knowledge and trends with accuracy and relevance. Additionally, a full-text filter was used. 

**Figure 1:**
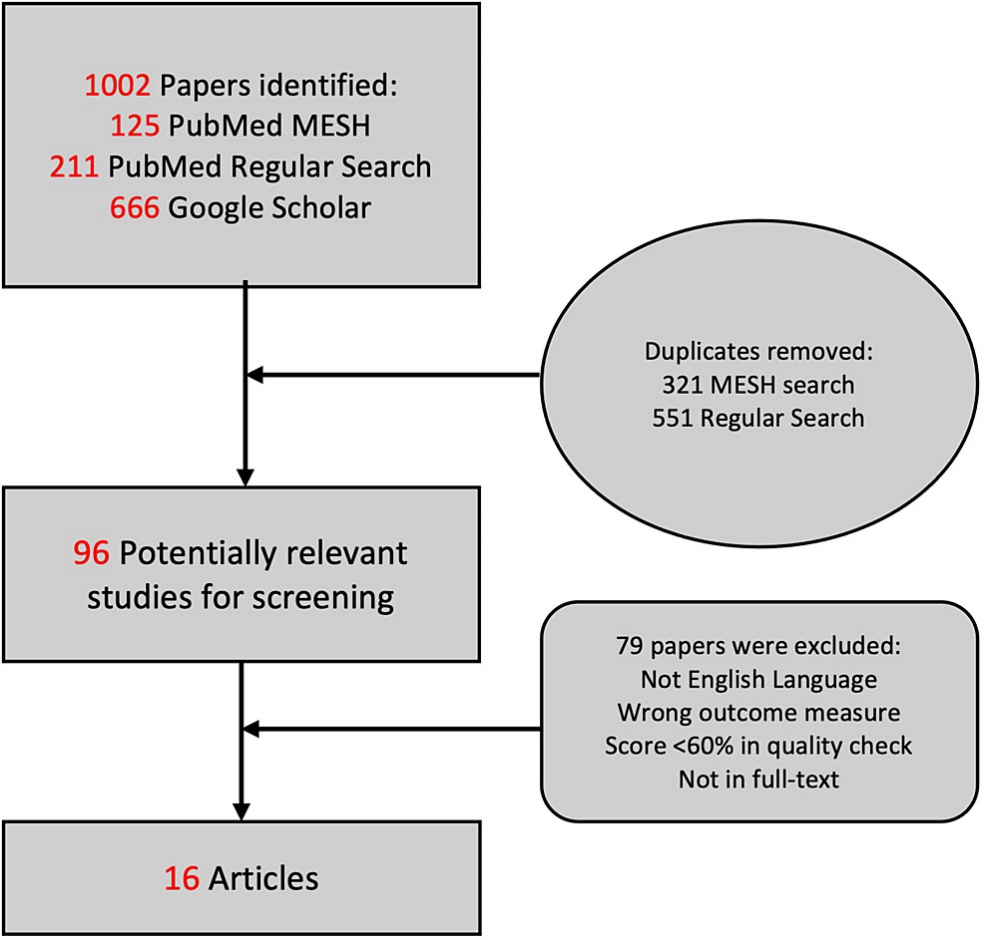
PRISMA flow diagram MESH: Medical Subject Heading

The search results were transferred into EndNote (Clarivate Plc, London, United Kingdom) and transformed into a Microsoft Excel (Microsoft Corporation, Redmond, Washington, United States) file as a second step. The duplicate papers were then removed using the duplicate filter. Following an analysis of the article titles, relevant titles were retained on a separate sheet. The quality of these papers was then evaluated using the following scales: the Newcastle-Ottawa Quality Assessment Scale (NCOS), the Scale for the Assessment of Narrative Review Articles (SANRA), and A MeaSurement Tool to Assess systematic Reviews (AMSTAR). Table 2 and Figure 1 summarise the results of the entire process.

**Table 2:** Results of the full research process MESH: Medical Subject Headings

Search engine	Search strategy	Papers identified	Picking relevant titles	Post-scanning relevant abstract	Post quality check
PubMed	MESH	125	48	26	13
PubMed	Regular (systematic review)	211	45	8	3
Google Scholar	Advanced search	666	3	0	0
Total		1002	96	37	16

Results

Our systemic search yielded a total of 1002 papers, and after detailed article screening, a total of 16 articles were included in this research. Out of these, there were eight non-randomised clinical trials (NRCT), three system reviews, three case series studies, two cohort studies, and one undefined research paper. A quality appraisal check was then performed to score these papers.

The NCOS is shown in Table 3 [7].

**Table 3:** Quality appraisal (Newcastle-Ottawa Quality Assessment Scale (NCOS): non-randomised clinical trials/cohort study) [2,4-5,7-16]

	Baser et al. [8]	Bektas et al. [9]	Bertossi et al. [10]	De Rosa et al. [11]	Esen et al. [5]	Giammarioli and Liberti [2]	He et al. [12]	Josipovic et al. [13]	Jung et al. [14]	Ramos et al. [15]	Santorelli and Marlino [4]	Trivisonno et al. [16]
Representativeness of the exposed cohort	1	1	1	1	1	1	1	1	1	1	1	1
Selection of the non-exposed cohort	0	0	0	0	0	0	0	0	0	0	0	0
Ascertainment of exposure	1	1	1	1	1	1	1	1	1	1	1	1
Demonstration that outcome of interest was not present at the start of the study	1	1	1	1	1	1	1	1	1	1	1	1
Comparability of cohorts based on the design or analysis	1	1	1	1	0	1	0	1	0	0	1	1
Assessment of outcome	1	1	1	1	1	1	1	1	1	1	1	1
Was follow-up long enough for outcomes to occur?	1	1	1	1	1	1	1	1	1	1	1	1
Adequacy of follow-up of cohorts	1	1	1	1	1	1	1	1	1	1	1	1
Total Score	7	7	7	7	6	7	6	7	6	6	7	7

AMSTER Quality Appraisal Tool results are shown in Table 4 below [17]. 

**Table 4:** Quality appraisal (Assessment of Multiple Systematic Reviews (AMSTAR)) [3,17-19] PICO: population, intervention, control, and outcomes; RoB: Risk of Bias

	Beneduce et al. [3]	Bouaoud and Belloc [18]	Mortada et al. [19]
Did the research questions and inclusion criteria for the review include the components of PICO?	No	Yes	Yes
Did the report of the review contain an explicit statement that the review methods were established prior to the conduct of the review and did the report justify any significant deviations from the protocol?	No	Yes	Partial Yes
Did the review authors explain their selection of the study designs for inclusion in the review?	Yes	Yes	Yes
Did the review authors use a comprehensive literature search strategy?	Partial Yes	Yes	Yes
Did the review authors perform study selection in duplicate?	Yes	Yes	Yes
Did the review authors perform data extraction in duplicate?	Yes	Yes	Yes
Did the review authors provide a list of excluded studies and justify the exclusions?	No	No	No
Did the review authors describe the included studies in adequate detail?	No	Yes	Yes
Did the review authors use a satisfactory technique for assessing the RoB in individual studies that were included in the review?	No	No	No
Did the review authors report on the sources of funding for the studies included in the review?	Yes	Yes	Yes
If meta-analysis was performed did the review authors use appropriate methods for statistical combination of results?	Not Applicable	Not Applicable	Not Applicable
If meta-analysis was performed, did the review authors assess the potential impact of RoB in individual studies on the results of the meta-analysis or other evidence synthesis?	Not Applicable	Not Applicable	Not Applicable
Did the review authors account for RoB in individual studies when interpreting/ discussing the results of the review?	No	Yes	Yes
Did the review authors provide a satisfactory explanation for, and discussion of, any heterogeneity observed in the results of the review?	No	No	Yes
If they performed quantitative synthesis did the review authors carry out an adequate investigation of publication bias (small study bias) and discuss its likely impact on the results of the review?	Not Applicable	Not Applicable	Not Applicable
Did the review authors report any potential sources of conflict of interest, including any funding they received for conducting the review?	Yes	Yes	Yes

In the paper by Raggio and Asaria, quality was assessed using the SANRA tool with a total score of 9 [20,21]. 

Across all the included studies, 652 patients were identified and included. All these patients had undergone NSR treatment. A total of 93% of those patients have a satisfactory result. The other 7% experienced low satisfaction with NSR due to minor complications including bruising, pain, irregularity, and an uneven surface of the nasal dorsum. A summary of the characteristics of all articles included is provided in Table 5. 

**Table 5:** Included articles' characteristics. [2-5,8-16,18-20] NRCT: Non-randomised clinical trial; N/A: Not applicable; HA: Hyaluronic acid; NSR: Non-surgical rhinoplasty; SR: Surgical rhinoplasty

Study reference	Type of study	Number of papers reviewed if applicable	Number of patients included if applicable	Patients’ satisfaction with NSR	Main complications with the use of NSR	The main outcome of the research
Baser et al. [8]	NRCT	N/A	20	High	None	The study illustrates the use of NSR in treating patients with nose imperfections, emphasizing the use of HA fillers for NSR over other agents due to their safe profile and reversible results. The study also highlights the importance of using the correct technique when injecting fillers into the nose to prevent adverse effects associated with inadequate treatment.
Bektas et al. [9]	Case-series study	N/A	85	90.5%	1) Redness and vascular impairment	NSR is a viable alternative to SR for patients who decline surgical procedures. HA fillers are the preferred method for NSR because they can be readily dissolved if patients are dissatisfied with the results.
Beneduce et al. [3]	Systematic review study	14	N/A	N/A	N/A	The duration of NSR's effects is intricate and can vary among individuals, necessitating further studies for a comprehensive understanding.
Bertossi et al. [10]	NRCT	N/A	107	High	Redness and swelling, infection, and lumps	NSR is a safe, efficient, and cost-effective alternative to SR for treating minor nasal abnormalities like a pronounced hump, under-projected tip, nasal depressions, saddle nose, and trauma-related disorders. NSR often results in great patient satisfaction and can produce effects that persist between eight months and a year. Patients are recommended to consider repeating the operation around one year later to maintain the desired outcomes and assure sustained pleasure.
Bouaoud and Belloc [18]	Systematic review study	15	N/A	80-100%	Skin necrosis and severe bruising	Surgery continues to be the gold standard for nose correction procedures. However, it can be concluded that NSR can complement SR, potentially reducing costs, procedure duration, and long-term adverse effects.
De Rosa et al. [11]	Cohort study	N/A	74	High	Haematoma, granuloma, and swellings	NSR serves as a favourable alternative to SR, achieving high patient satisfaction with results lasting up to one year post-treatment. Furthermore, the findings indicate that NSR is not associated with significant adverse effects.
Esen et al. [5]	NRCT	N/A	40	High	Skin necrosis, supratip deformity and irregular outline if the area overlying the dorsal cartilage is overfilled, oedema, erythema, pain, and bruising	NSR successfully enhanced minor nasal deformities and improved the quality of life for patients. This study also delved into various types of fillers, outlining their advantages and disadvantages when administered around the nose. Additionally, the study provided insights into diverse techniques applicable to filler treatments around the nose.
Giammarioli and Liberti [2]	NRCT	N/A	101	84.2%	Infection, mild oedema, and bruising	The study results have demonstrated that NSR is an effective and safe procedure, leading to high patient satisfaction. Nevertheless, further research is necessary to evaluate its long-term outcomes.
He et al. [12]	Cohort study	N/A	46	Low	Surface irregularity of the nose, redness and swelling, and depression	Improper injection of NSR can impose a significant psychological burden on patients' lives. Those who subsequently underwent surgical curettage to remove the improperly injected material reported satisfaction and experienced an improved quality of life.
Josipovic et al. [13]	NRCT	N/A	20	High	Haematoma	This study focused on the five-point liquid rhinoplasty technique. The findings revealed that NSR using this specific technique is a fast, efficient, and safe procedure, offering a viable alternative to surgery. Additionally, the study demonstrated that the outcomes of this procedure typically hinge on the depth of injection and the positioning of four out of the five injection points along the midline.
Jung et al. [14]	NRCT	N/A	20	N/A	N/A	Direct percutaneous injection from the glabella can improve the precision of sellion filler augmentation rhinoplasty, potentially lowering the risk of problems like vision loss and skin necrosis due to vascular compromise.
Mortada et al. [19]	Systematic review study	23	N/A	75-98%	Bruising and pain. The uneven surface of the nasal dorsum	NSR is associated with minimal side effects and requires a short healing period. Furthermore, it has consistently led to excellent patient satisfaction.
Raggio and Asaria [20]	Research paper	N/A	N/A	N/A	N/A	This paper highlights the advantages of employing NSR as a substitute for SR. The study also delves into various techniques employed in this form of treatment. Additionally, the review addresses the limitations of NSR, particularly its effectiveness in cases involving patients with severe dorsal humps, deviation, and tip rotation, as these individuals may not derive significant benefits from NSR.
Ramos et al. [15]	Case-series study	N/A	3 cases	N/A	N/A	Patients who opt for NSR and later decide to undergo SR must choose between waiting for the fillers to naturally reabsorb, utilizing hyaluronidase to dissolve the fillers, or proceeding with SR directly if the fillers cannot be reabsorbed.
Santorelli and Marlino [4]	NRCT	N/A	62	>80%	Pain, oedema, and haematoma in the dorsum of the nose	The treatment with 1 ml of HA fillers yielded high patient satisfaction and produced favourable results.
Trivisonno et al. [16]	Observational case-series study	N/A	14	80%	No major side effects	This study concentrated on the utilization of fluid cartilage as a form of NSR, demonstrating its effectiveness in addressing minor irregularities of the nose dorsum with minimal side effects.

Discussion

The advent of NSR, also known as the liquid nose job, has completely changed the field of aesthetic medicine, and it is gaining lots of interest and popularity among patients. Dermal filler injections are used to contour and improve the appearance of the nose. These non-surgical techniques for rhinoplasty have many benefits over traditional SR, including a shorter recovery period, reduced complication risks, and the potential for fast, reversible results. However, the growing popularity of NSR also prompts significant concerns and questions among patients and physicians in regard to their safety, efficiency, and long-term adverse effects. This article discusses the growing acceptance of NSR, as well as its safety, efficiency, satisfaction of patients, and wider implications for the practice of aesthetic medicine. This article aims to provide a thorough grasp of NSR as an appealing option for people seeking nasal enhancement and face harmony by reviewing and analysing the 16 articles included in this research paper.

Giammarioli and Liberti's NRCT study included 101 patients who had undergone NSR with 25 mg/mL HA fillers [2]. The study results showed that 84.2% of patients were very satisfied with the natural and predictable results of the procedure. The study focused on the importance of choosing the right HA concentration and product, as well as the right technique, to achieve a satisfactory reversible result. The trial also concluded that although the longevity of the results may vary from one patient to another, most HA fillers should last up to 12 months. This study had limitations, including being an open-label design, which could have caused an underreporting of adverse effects. Moreover, 25 mg/mL is a low amount of filler that is less likely to give unsatisfactory results [2]. Yet, patient satisfaction was also marked by the Santorelli & Marlino NRCT and Bertossi, et al. studies [4,10]. 

Additionally, a system review by Mortada, et al. that included 23 papers showed that the use of hyaluronic acid (HA) fillers for NSR resulted in high patient satisfaction and a low rate of complications [19]. Furthermore, the study also noted that although NSR can be a good replacement for SR, it may not be suitable for all cases as its uses are limited to fixing and reshaping minor nasal deformities and dorsal irregularities [19]. Furthermore, He et al. stated that improper injection can lead to serious implications for patients' physical and psychological health, and it was found that some patients required surgical curettage to remove the failed injected material to achieve satisfaction and a better quality of life [12]. Thus, in Beneduce et al.'s systemic review paper, it was stated that due to the complexity associated with the longevity of NSR, further research is required regarding this field [3].

Since the evolution of NSR, continuous research and studies have aimed to find the perfect technique and procedure with the most minimal side effects. Jung et al. have shown that although superficial injections of the nose result in a more effective augmentation, deeper injections are safer in limiting blood vessel compression since the vessels are thicker and stronger in the deeper layers. Moreover, the study also emphasises the use of epinephrine along with fillers to compress the blood vessels in the nose, thus limiting the chances of vascular complications [14]. However, Esen et al.'s NRCT stated that skin necrosis due to nasal fillers usually occurs because of the use of products that cannot be biodegraded [5]. The study also supported rubbing the area should injection into a vessel occur, thus allowing the dissolution of the polymers. A second technique to dissolve the injected material could include using hyaluronidase [5]. Another NRCT study by Josipovic et al., which involved 20 patients, discussed the effective use of five-point liquid rhinoplasty [13]. The study illustrated that the following method is a simple and effective technique that can limit and minimise complications [13]. The study illustrated that the results of this procedure usually depend on the depth of the injection and the position of four of the five injection points within the midline. The results of the study suggested vascular damage complications may be limited by an injection in the supraperiosteal plane, above the cartilage, or an injection to the midline. The study also supports the use of aspiration before the injection of fillers as a safety checkpoint to avoid intravascular occlusion [13].

The Josipovic et al. study had limitations that may raise the risk of bias and challenge the accuracy of the results of this paper. These limitations include the study being conducted only on Caucasian individuals, which limits the results to this racial group; moreover, it was a single-centre analysis based on a small sample size [13]. On the other hand, another paper by Trivisonno et al. focused on the use of fluid cartilage as a type of nasal filler to fix and repair minor irregularities in the nose dorsum [16]. However, the disadvantage of this technique is that cartilage may be lost in the dead space of the needle, and there is no estimate of the percentage of cartilage survival, indicating that further procedures may be needed for better result satisfaction. Furthermore, this procedure is lengthy and requires a pre-SR for the collection of shaved cartilage from the nasal septum, thus limiting the flexibility of the technique in general [16]. HA fillers remain the most advised nasal fillers due to their reversible, quick, and safe profile. Baser et al. stressed the importance of picking the right fillers and the right technique to avoid and limit adverse effects [8].

Likewise, with every new procedure, there are limitations associated with it. A study by Di Rosa et al. assessed the psychological outcomes of patients undergoing NSR through a questionnaire review given to patients one year post-NSR [11]. The analysis of the study showed an improvement in patients’ perception of their noses post-NSR. However, as expected, the study proved that patients reported no change in breathing, stressing the fact that NSR can alter external features of the nose, yet it has no effect on extreme nasal deformities that limit patients' breathing and comfort. The results of the questionnaire also showed that despite the high satisfaction reported by patients, these patients still had not disregarded the idea of further surgical procedures in the future [11]. Furthermore, NSR has no rule in the fixation of severe dorsal humps, deviation, and tip rotation as these problems can’t be treated without surgical intervention [20].

In comparison, the Bektas et al. case-series study, which included 85 patients in total, focused on the use of nasal filling procedures alongside rhinoplasty to alter and fix post-rhinoplasty defects [9]. The study also reviewed the safety of using dissolvent agents in dissatisfied NSR patients before surgical fixation. Results showed high satisfaction among patients who had undergone HA fillers post-rhinoplasty. Also, satisfactory and safe results were observed among those who dissolved their fillers at least one week before surgical fixation, concluding that both procedures can be used together to aim for high patient satisfaction [9]. Ramos et al.'s case series and Bouaoud and Belloc's systematic review also both supported the use of dissolvent before SR in patients with previous fillers [15,18].

This systematic review does have several limitations that should be acknowledged. First, none of the studies included in the review were randomised control trials (RCTs), which are widely regarded as the gold standard in research for providing the highest level of evidence. This absence of RCTs limits the generalizability of the results. Second, the NRCTs that were analysed had relatively small sample sizes, potentially reducing their representativeness for the broader population. Third, there was a notable lack of objective data comparing SR to NSR directly, which could have provided more robust insights. Fourth, the follow-up periods in the NRCT studies were relatively short, which restricted the assessment of long-term side effects and complications associated with NSR. Finally, some of the studies included specific ethnic groups, which may limit the applicability of the findings to populations beyond those specific ethnic origins. These limitations should be considered when interpreting the results of this review.

## Conclusions

NSR presents a promising alternative to traditional surgical nose procedures, offering a non-invasive approach that involves the use of dermal fillers to enhance and reshape the nose's appearance. This method has gained popularity among individuals seeking subtle yet effective changes without the typical downtime and surgical risks. This review highlights NSR as a swift, secure, and efficient substitute for SR. The existing body of literature generally supports the use of HA fillers for correcting minor defects in the nasal dorsum. Nevertheless, it's crucial to recognise that the risks and potential limitations of this procedure continue to be debated, primarily because the follow-up periods in NRCT papers included were relatively brief, constraining the evaluation of long-term side effects and complications linked to NSR. Therefore, the included research underscores NSR as a distinct treatment option for individuals with modest concerns about their nose shape rather than a direct replacement for surgical rhinoplasty. To advance our understanding and standardise this increasingly popular but still evolving field, it is imperative to conduct an RCT study utilising photographic evidence. Such a study could contribute significantly to establishing NSR's role in cosmetic procedures and ensuring its safety and efficacy in meeting patient expectations.
